# Performance analysis of the taekwondo pushing kick (Miluh-chagi) in adolescent black belts

**DOI:** 10.3389/fspor.2026.1835775

**Published:** 2026-07-06

**Authors:** José Luís Sousa, Victor Hernández-Beltrán, Mario C. Espada, Hugo Louro, José M. Gamonales

**Affiliations:** 1 Research Group in Optimization of Training and Performance Sports, Faculty of Sport Science, University of Extremadura, Cáceres, Spain; 2 European Taekwondo Union, Education and Applied Science Committee, Attendorn, Germany; 3 Sport Sciences School of Rio Maior, Polytechnic Institute of Santarém, Rio Maior, Portugal; 4 Escola Superior de Educação, Instituto Politécnico de Setúbal (CIEQV Setúbal), Setúbal, Portugal; 5 SPRINT Sport Physical Activity and Health Research & Innovation Center, (SPRINT), Sport Sciences School of Rio Maior, Polytechnic Institute of Santarém, Rio Maior, Portugal; 6 Comprehensive Health Research Centre (CHRC), Universidade de Évora, Évora, Portugal; 7 Centre for the Study of Human Performance (CIPER), Faculdade de Motricidade Humana, Universidade de Lisboa, Oeiras, Portugal; 8 Faculty of Education and Psychology, University of Extremadura, Badajoz, Spain; 9 Instituto Universitario de Investigación e Innovación en el Deporte (INIDE), Universidad de Extremadura, Cáceres, España

**Keywords:** adolescent athletes, martial arts, observational reliability, performance analysis, sport combat

## Abstract

**Introduction:**

Taekwondo, a Korean martial art and Olympic sport since the Sydney 2000 Olympic Games, emphasizes kicking skills for optimal performance. The pushing kick (Miluh-chagi) targets the opponent's midsection or head and follows a linear path. It is a standard push kick in Taekwondo and can score points like other techniques. This study aimed to analyse the technical performance indicators during the execution of the pushing kick (lifecycles of ten kicks) by Taekwondo athletes, using an observational tool: the Observation System for Technical Performance Indicators—Chagi (OSTIP-C).

**Methods:**

This study used observational methods to assess motor competence in adolescent athletes during a pushing kick, analysing a total of 12 athletes (5 females, 7 males). The reliability and precision of scores were assessed across 120 kicking trials, consisting of ten execution lifecycles performed by each of the twelve taekwondo black belt athletes in the sample. Precision was measured with the coefficient of variation, and evaluator reliability with the intraclass correlation coefficient. Student's *t*-test compared maximum variable values, while Spearman's rho analyzed correlations. In the same line, Linear Mixed-Effects Model were used to analyze data where observations are not independent (e.g., repeated measures of the lifecycle of the Miluh-chagi Taekwondo kick). It decomposes variance into fixed effects (lifecycle of the kick) and random intercept (performance score by subjects). The level of significance was established on *p* < 0.05.

**Results:**

The sample data were reliable, with strong intra- and inter-rater agreement. Significant differences appeared in foot take-off, knee up, and start leg flexion (*p* < 0.05; Cohen's *d* = 0.8). Conduct and aggregate criteria showed small to moderate negative correlations (*r* = 0.181; *p* < 0.05 and *r* = 0.396; *p* < 0.01). On the other hand, there was a perfect correlation between support leg position and attacking leg foot position.

**Discussion:**

The results show significant differences in the foot take-off, knee-up, and start-leg flexion phases, highlighting these early moments of the movement as the most variable and technically demanding. These phases appear to be critical for the coordination and efficiency of the kicking action.

## 1 Introduction

Taekwondo is a Korean martial art and combat sport that has been an Olympic discipline since the Sydney (2000) Olympic Games. This combat sport originated in medieval cultures; today, it is primarily a combat activity that is performed under safety competition rules with dynamic movements, including kicks, postural shifts, and coordinated footwork (1–3). In Taekwondo, kicking skills are crucial for high motor performance. The pushing kick (*Miluh-chagi* in Korean) is a Taekwondo kick technique aimed at the opponent's midsection (body armor gear) or head (head gear) (4). It is a typical Taekwondo push kick, but it can also score like any other technique (kick or punch). The trajectory follows a linear movement. The foot contact with the target can be made with the tip of the foot with flexion of the toe, the outside of the foot, and the sole of the foot (2). Taekwondo athletes can successfully use this kick with the front leg, easily change the target zone, and correct the trajectory with an advantageous form; this movement needs less time relative to speed for foot motor competence (5, 6). It is an individual sport that is classified as requiring a “recognition reaction time”, where right laterality is significantly more important than left laterality (7) so that the individual can respond quickly to the opponent's moves. Consequently, performance in this technical action seems to be determined by both biomechanical execution factors and perceptual–motor processes related to rapid decision-making in combat situations.

In Taekwondo, four phases for kicks [Start (A), Toe Off (B), Maximum Knee Flexion (C), and Impact (D)] and three moments (Push, Lift, and Strike) have been identified (5, 6, 8, 9). Studies have emphasized that all this movement must be executed at high velocity through rapid pelvic axial rotation, hip abduction, hip flexion, and knee extension, combined with rapid movement of the center of mass toward the target. On the other hand, authors such as Gavacan et al. (10), and Barnamehei and Safaei (11) have defined three phases and six moments: foot take-off, knee up, contact moment, start leg extension, start leg flexion, and thigh extension, in which the perfect execution of the athletes' kicking skills is essential for good motor performance. In Taekwondo, a combat sport, the outcome depends, for the most part, on the technical perfection of the kick, where the athlete's execution is essential for good performance and a master-level presentation of the martial art (11).

Taekwondo coaches believe that they have a “magical method or eagle eye”, and that is why they feel comfortable analyzing athletes' skills without using tools or devices. O'Donoghue (12) argued that the role of a motor performance analyst working with coaches and athletes is to enhance performance through a cycle of observation, analysis, reflection, planning, and action, and to compare the athlete's performance with the ideal performance. The development and implementation of new measurement technologies (e.g., observational video analysis systems) with multiple practical applications have intensified the focus on the analysis of adolescent motor performance in sport (12, 13). Researchers have shown that human observation and memory are not reliable enough to provide accurate and objective information for high-performance athletes and measuring tools using technology and multidisciplinary research into the Taekwondo Olympic discipline are necessary to enable an effective feedback process (11, 13–15). Consequently, the use of objective measurement tools and technological systems has become essential to ensure accurate performance analysis and to enhance the feedback process in taekwondo.

Adopting the framework of Frank and Goodman (16), our study focuses on individual technique analysis through a systematic five-step process: (1) identifying technical behaviors in specific offensive and defensive context; (2) cataloging sequentially timed events with associated success/failure ratios; (3) data tabulation for objective profiling; (4) generating athlete-specific technical feedback; and (5) facilitating professional coaching to inform future interventions.

Observational methods make it possible to analyze situational elements in taekwondo, assess visible technical and tactical actions, and uncover relationships like frequency, sequence, and timing (17). This approach ultimately helps identify patterns of behavior and the relationships between key behaviors (focal and conditioned), offering the information needed for decision-making (17, 18). Thus, this study used an observation methodology for sport behavior, codification, and data collection and analysis that is used in physical and sports sciences (19, 20). In this study, the recent and comprehensive proposal by Sousa et al. (2) is followed, which comprises three phases, six main observation moments, forty-eight conduct-and-aggregate criteria, and 153 alphanumeric codes, supported by a validated observation unit. This methodological framework provides a rigorous and systematic approach to the analysis of technical–tactical behaviors in taekwondo, enabling a deeper understanding of the interaction patterns that emerge during combat.

While Taekwondo techniques have been extensively studied, there is limited research that systematically examined the biomechanical phases of the pushing kick, particularly through an observational methodology. It is hypothesized that the technical execution of this kick would vary significantly between phases, with higher intra- and inter-rater agreement for critical contact moments. Across all metrics, the current study yielded excellent to near-perfect reliability, representing an advancement over the results reported by Sousa et al. (9). This study aimed to examine the technical performance indicators observed during Taekwondo athletes' execution of the pushing kick, assessed over the lifecycles of ten consecutive kicks. This study used a previously designed, validated, and published observation tool: the Observation System for Technical Performance Indicators—Chagi (OSTIP-C) (2).

## 2 Materials and methods

### 2.1 Study design

The present study followed a quantitative empirical design using an arbitrary observation code in a natural training environment (21). The observational methodology of Anguera and Hernández-Mendo (19) was applied, following a nomothetic and multidimensional design. Motor competence was assessed during a single session, comparing different integral units of analysis (Miluh-chagi lifecycles) and multiple response levels.

To achieve the study objectives, the OSTIP-C instrument was used (2). This instrument includes three phases, six observation moments, and a comprehensive set of conduct and aggregate criteria. Evaluators coded athletes' motor competence events into 10 technical gestures.

### 2.2 Study variables and conduct and aggregate criteria

The foot take-off (V1), knee up (V2), start leg extension (V3), contact moment (V4), start of leg flexion (V5), and thigh extension (V6) variables were considered the characteristic movements, which were subdivided based on conduct criteria (AL—attacking leg and attacking leg's foot position and SL—support leg and support leg's foot position), aggregate criteria (H—head; T—trunk; La—left arm and forearm; and Ra—right arm and forearm position), and 153 alphanumeric codes.

### 2.3 Data on the lifecycle of ten kicks and the athlete sample

The sample consisted of 12 adolescent Portuguese Taekwondo athletes (5 females, 7 males) who each performed 10 kicks, resulting in 120 kick lifecycles. The athletes' characteristics (mean ± SD) were:

- Age: 15.5 ± 2.6 years.
- Height: 160.3 ± 4.4 cm.
- Weight: 55.3 ± 7.5 kg.
- Years of practice: 6.3 ± 1.3 years.

Regarding belt rank, 8 athletes held a 1st DAN black belt, and 4 held a 2nd DAN black belt. All athletes were members of the Portuguese national team and had competed in at least one international competition. Accordingly, World Taekwondo (22), the official weight categories are: male (U45 kg, U48 kg, U51 kg, U55 kg, U59 kg, U63 kg, U68 kg, U73 kg, U78 kg, and +78 kg), and female (42 kg, U44 kg, U46 kg, U49 kg, U52 kg, U55 kg, U59 kg, U63 kg, U68 kg, and +68 kg).

### 2.4 Inclusion and exclusion criteria

Athletes were eligible for participation if they met the following inclusion criteria: (1) provided written informed consent; (2) were in good health; (3) were members of the Portuguese national team at least once; (4) had a Taekwondo black belt rank; and (5) were teenagers. The exclusion criteria were as follows: (1) has a mental health disorder, (2) is currently using medication, and (3) did not provide a fully completed informed consent form.

### 2.5 Evaluator panel

All evaluators received prior, strict observation training in accordance with the methodology's recommendations Anguera & Hernández-Mendo (19) and Anguera et al., (20). A suitable observer and evaluator must learn “to see what they are really asked to see” (19, 20). The methodological recommendations proposed by Anguera et al. (20) to train the evaluators were used. The evaluators underwent a 5-h calibration session in which they practiced coding kick executions using the OSTIP-C system; they achieved >85% agreement before beginning data collection for this study. The evaluator panel consisted of five Taekwondo experts:

- Evaluator 1 was an international expert with more than 35 years of experience and is a national G3-level coach, a world-level coach, and a 7th DAN black belt.
- Evaluator 2 has over 30 years of experience and is a European coach and a 6th DAN black belt.
- Evaluators 3 and 4 have more than 20 years of experience and are national G3-level coaches and 5th DAN black belt.
- Evaluator 5 has over 15 years of experience and is a national G2-level coach and 4th DAN black belt.

DAN refers to advanced martial arts ranks, which indicate superior applied knowledge and skills.

### 2.6 Procedure

The data collection was conducted in a single day in Rio Maior City, Portugal, on February 17, 2025. All the measurements were conducted between 8:00 and 10:00 AM to minimize diurnal variations in motor competence. First, the athletes were informed of the procedures and purpose of the study and asked to fill out an informed consent form. They then received a briefing session on the conditions under which the protocol would be performed, the objectives of the study, and the measurements that would be taken. The execution of the technical gesture consisted of ten kicks at maximum speed without an oral or whistle command. For data collection, the procedure proposed by (2, 9) was followed: To minimize optical distortions, perspective errors, and parallax anomalies inherent to 2D video captures, the high-definition camcorder was mounted at a fixed height of 1.2 m, positioned strictly orthogonal (90°) relative to the athletes' plane of action, and placed exactly 500 cm from the execution trajectory line. Execution paths were visually standardized via floor markings. This setup, combined with rigorous multi-hour evaluator training, yielded high intra- and inter-rater consistency (ICC > 0.94), verifying that multi-planar actions did not introduce classification bias. The adolescent athletes performed ten kicks (lifecycles) to a boxing bag, and they were all requested to act with their dominant leg.

A Casio ZR200 high-definition video camera with 10x Optical Zoom [frequency: 50 Hz; focal length: 52 mm; sensitivity: ISO 400; aperture: F3.0 (W) to F7.9 (W); and shutter time: 1.80] was used to record the images/videos. Match Vision Software was used to capture digital images and view the videos in AVI format. Kinovea software (version 0.9.5) was used strictly as a specialized video playback interface to optimize visual analysis (e.g., frame-by-frame scraping and slow-motion tracking) to support the human-expert categorization of technical indicators within the OSTIP-C framework. This software has been used in sports sciences and clinical research (23). After collecting images of 10 kick lifecycles performed by the adolescent athletes, the evaluator panel conducted the training, testing, retesting (carried out 15 days later), and coding phases.

### 2.7 Statistical analysis

A power analysis (GPower 3.1) determined that a minimum of 10 participants was required to detect significant differences in technical performance (*α* = 0.05; *power* = 0.80). The method outlined in (24) was followed. The results were presented as the mean ± Standard Deviation (SD). The Intraclass Correlation Coefficient (ICC) between observers was used to assess evaluators' reliability; the values varied across variables and observation moments. ICC estimates and their 95% confidence intervals were calculated based on a mean-rating (*k* = 5), absolute-agreement, 2-way mixed-effects model. The reference values for the ICC were <0.5 (poor), 0.5–0.75 (moderate), 0.75–0.90 (good), and ≥0.90 (excellent). The Standard Error of Measurement (SEM) estimates the variation around an individual's true score based on repeated measures; it was calculated using the formula SEM = SD * sqrt(1 − *r*), where SD is the standard deviation and r is the reliability. The Coefficient of Variation (CV) is the ratio of the SD to the mean and indicates the extent of variability relative to the population mean. The CV was expressed as a percentage [CV = (SD/Ẋ) × 100] and was used to verify the precision (25–28).

To assess the differences in the maximum values of each variable, Student's *t*-test was used. This statistical test allowed for the comparison of sample means, providing insight into whether the observed maximum values were significantly different across the variables examined. The effect size (ES—Cohen's *d*) was calculated and rated according to (27–31), as |*d*| ≥ 0.80 (large). The criteria of Hopkins et al. (26) were used to assess the strength of the correlations: 0–0.30 (small), 0.31–0.49 (moderate), 0.50–0.69 (large), 0.70–0.89 (very large), and 0.90–1 (almost perfect). Correlation coefficient analysis was carried out using Spearman's rho, a nonparametric test, because the continuous variables did not meet the assumption of normality based on the Kolmogorov–Smirnov test. The correlation coefficients for all variables did not exceed 0.85, indicating no multicollinearity (25, 26, 32). Categorical alphanumeric codes from the tool (OSTIP-C) were converted into an ordinal numeric scale (e.g., 1 = 1CL1 − In trunk extension) to permit the calculation of mean scores and the use of parametric inferential statistics. All data analyses were performed using IBM SPSS Statistics, version 24.0 (SPSS Inc., Chicago, IL), and the figures were created in Excel for Windows. Statistical significance was set at *p* < 0.01 (**) and *p* < 0.05 (*) for all analyses in this study.

A Linear Mixed-Effects Model (LMM) examines how a response variable relates to explanatory variables, considering both fixed and random factors. LMMs use Likelihood-Ratio Tests (LRTs) to compare models and assess term significance. Researchers should clearly separate hypothesis testing from exploratory analysis to prevent Type I errors and reproducibility problems (33, 34). For all six variables with their conduct and aggregate criteria, a Linear Mixed-Effects Model (LMM) were used to analyze data where observations are not independent (e.g., repeated measures of the lifecycle of the Miluh-chagi Taekwondo kick). It decomposes variance into fixed effects (lifecycle of the kick) and random intercept (performance score by subjects). To account for the non-independence of the repeated-measures data (10 kicks nested within individual athletes), Linear Mixed-Effects Models (LMM) were applied. The subject factor was introduced as a random intercept to isolate individual variance. Fixed effects mapped the technical progression metrics across the individual kick's lifecycle. Converting categories into ordered numbers (1, 2, 3) gives the LMM a clear direction of “improvement” or “deviation” to calculate variance over the ten-kicks lifecycle per athlete. The assumptions of residual normality and homoscedasticity were verified and confirmed via visual inspections of residual-vs.-execution performance plots and normal Q-Q plots, exhibiting uniform linear scatter (35).

### 2.8 Ethical approval

The study adhered to the Declaration of Helsinki and was approved by the Bioethics and Biosafety Committee of the University of Extremadura (approval number: 79/2022, 16/06/2022). All athletes were informed of the study's nature and objectives and signed informed consent forms. Participation was voluntary, and athletes were free to withdraw at any time. Also, this investigation was developed under the Ethical Standards in Sport and Exercise Science Research (36). The investigation respected the framework of Organic Law 3/2018 of 5 December on Personal Data Protection and the guarantee of digital rights.

## 3 Results

Valid and complete results were obtained throughout the technical lifecycle of the 10 kicks performed by 12 athletes. To assess data quality, the coefficient of variation and intraclass correlation coefficient were calculated to confirm the reliability of the evaluator panel. Table 1 presents the ICC results, which indicate that the evaluators' reliability was good (*p* < 0.01).

**Table 1:** Intraclass correlation coefficient (ICC), Mean ± standard deviation (SD), standard erros of measurement, and coefficient of variation and confidence interval.

	Inter-rater ICC (95% CI)	Mean	SD	SEM	CV (95% CI)
Evaluator 1	0.956 (0.951–0.961)	960.00	7.07	0.05	3.96
Evaluator 2	0.813 (0.809–0.817)	813.00	5.65	0.04	4.36
Evaluator 3	0.821 (0.780–0.862)	821.00	57.98	0.04	3.93
Evaluator 4	0.875 (0.831–0.919)	875.00	62.23	0.04	3.96
Evaluator 5	0.827 (0.786–0.868)	827.00	57.98	0.04	3.99

	Intra-rater ICC (95% CI)				
Evaluator 1 (pre-test)	0.952 (0.947–0.957)	952.00	7.07	0.05	4.30
Evaluator 1 (post-test)	0.964 (0.959–0.969)	964.00	7.07	0.05	4.22
Average	0.958* (0.952–0.964)	958.00	8.48	00.6	4.26

SD, standard deviation; SEM, standard error of measurement; CV, coefficient of variation; CI, confidence interval; ICC, intraclass correlation coefficient; %, percentage.

* *p* < 0.001.

The technical performance was categorized into three phases, six observation moments and 153 alphanumeric codes (spanning both conduct and aggregate criteria). Phase 1 (preparation) consists of foot take-off (V1) and knee lift (V2). Phase 2 (execution) covers the initial contact moment (V3) through to the completion of contact (V4). Phase 3 (recovery) involves the initiation of leg flexion (V5) and subsequent thigh extension (V6). The technical lifecycle of the 10 Miluh-chagi Taekwondo kick was highly reliable (typical errors of 0.9%). The plot of residual vs. execution performance of this 10-kicks test showed a uniform scatter that justified the use of a linear effect of execution performance by athletes.

The foot take-off and knee up (phase 1) results are reported in Table 2. The foot take-off showed statistically significant differences in the position of the attacking leg foot (*p* < 0.01, Cohen's *d* = 2.7, large ES), and the knee up showed statistically significant differences in the position of the attacking leg foot (*p* < 0.01, Cohen's *d* = 2.4, large ES).

**Table 2:** Phase 1: foot take-off and knee up variables. Mean ± standard deviation (SD), Student's *t*-test result, and ES for lifecycles of all ten kicks.

	Variable (conduct and aggregate criterion)	Kick lifecycle		*t*-Value	*p*-Value	ES
		Mean	SD			
Phase 1	V1 | Foot take-off					
	AL1—Attacking leg position	2.19	0.592	0.259	0.796	0.149
	AL2—Attacking leg foot position	1.56	0.554	4.811	**<0**.001**	2.777
	SL1—Support leg position	1.37	0.650	0.636	0.526	0.164
	SL2—Support leg foot position	1.50	0.571	1.260	0.210	0.727
	H1—Head position	1.47	0.566	1.827	0.070	1.054
	T1—Trunk position	1.30	0.461	0.401	0.689	0.231
	La1—Left arm and forearm position	1.47	0.502	0.491	0.624	0.283
	Ra1—Right arm and forearm position	1.48	0.502	0.798	0.426	0.460
	V2 | Knee up					
	AL3—Attacking leg position	2.02	0.709	0.308	0.758	0.177
	AL4—Attacking leg foot position	1.85	0.783	4.264	**<0**.001**	2.461
	SL3—Support leg position	1.49	0.505	0.214	0.831	0.123
	SL4—Support leg foot position	1.65	0.670	1.464	0.146	0.845
	H2—Head position	1.75	0.690	1.841	0.068	1.062
	T2—Trunk position	1.47	0.650	1.586	0.115	0.915
	La2—Left arm and forearm position	1.70	0.461	0.401	0.689	0.231
	Ra2—Right arm and forearm position	1.57	0.492	1.955	0.053	1.128

V1–V2, variables 1 and 2; SD, standard deviation; ES, effect size.

Statistically significant correlations are shown in bold.

** *p* < 0.01.

The start leg extension and contact moment (phase 2) results are reported in Table 3. The start leg extension and contact moments did not show any statistically significant differences.

**Table 3:** Phase 2: start leg extension and contact moment variables. Mean ± standard deviation (SD), Student's *t*-test result, and ES for lifecycles of all ten kicks.

	Variable (conduct and aggregate criterion)	Kick lifecycle		*t*-Value	*p*-Value	ES
		Mean	SD			
Phase 2	V3 | Start leg extension					
	AL5—Attacking leg position	2.35	0.670	1.464	0.146	0.845
	AL6—Attacking leg foot position	2.25	0.914	0.101	0.919	0.058
	SL5—Support leg position	1.63	0.487	0.636	0.526	0.367
	SL6—Support leg foot position	1.45	0.573	0.164	0.870	0.094
	H3—Head position	2.03	0.878	0.350	0.727	0.202
	T3—Trunk position	2.56	0.587	0.997	0.321	0.575
	La3—Left arm and forearm position	2.00	0.589	0.000	1.000	0.000
	Ra3—Right arm and forearm position	1.47	0.562	0.207	0.836	0.119
	V4 | Contact moment					
	AL7—Attacking leg position	1.64	0.642	0.680	0.498	0.392
	AL8—Attacking leg foot position (area)	2.92	2.110	0.617	0.539	0.356
	SL7—Support leg position	1.55	0.502	0.185	0.854	0.106
	SL8—Support leg foot position	2.80	1.475	1.467	0.145	0.846
	H4—Head position	1.95	0.849	0–109	0.914	0.062
	T4—Trunk position	2.56	0.546	1.212	0.228	0.699
	La4—Left arm and forearm position	2.01	0.437	1.661	0.099	0.958
	Ra4—Right arm and forearm position	1.60	0.774	0.485	0.628	0.280

V3–V4, variables 3 and 4; SD, standard deviation; ES, effect size.

The start leg flexion and thigh extension (phase 3) results are reported in Table 4. The start leg flexion moment showed a statistically significant difference in the support leg foot position (*p* < 0.01, Cohen's *d* = 1.8, large ES). The thigh extension moment did not show a statistically significant difference.

**Table 4:** Phase 3: start leg flexion and thigh extension variables. Mean ± standard deviation (SD), Student's *t*-test result, and ES for lifecycles of all ten kicks.

	Variable (conduct and aggregate criterion)	Kick lifecycle		*t*-Value	*p*-Value	ES
		Mean	SD			
Phase 3	V5 | Start leg flexion					
	AL9—Attacking leg position	1.97	0.921	0.067	0.947	0.038
	AL10—Attacking leg foot position	2.59	0.979	0.536	0.593	0.309
	SL9—Support leg position	1.67	0.586	1.303	0.195	0.752
	SL10—Support leg foot position	1.82	0.606	3.272	**<0**.001**	1.889
	H5—Head position	2.01	0.897	1.820	0.071	1.050
	T5—Trunk position	2.49	0.594	0.052	0.959	0.030
	La5—Left arm and forearm position	1.90	0.763	0.246	0.806	0.052
	Ra5—Right arm and forearm position	1.43	0.715	2.102	0.038	1.213
	V6 | Thigh extension					
	AL11—Attacking leg position	1.48	0.601	0.674	0.502	0.389
	AL12—Attacking leg foot position	1.98	0.653	0.332	0.740	0.191
	SL11—Support leg position	1.43	0.568	0.577	0.565	0.333
	SL12—Support leg foot position	1.98	0.653	0.332	0.740	0.191
	H6—Head position	1.91	0.903	0.784	0.434	0.452
	T6—Trunk position	2.23	0.701	0.977	0.331	0.564
	La6—Left arm and forearm position	1.51	0.679	1.035	0.303	0.597
	Ra6—Right arm and forearm position	1.70	0.912	0.781	0.436	0.502

V5–V6, variables 5 and 6; SD, standard deviation; ES, effect size.

Statistically significant correlations are shown in bold.

** *p* < 0.01.

**Figure 1:**
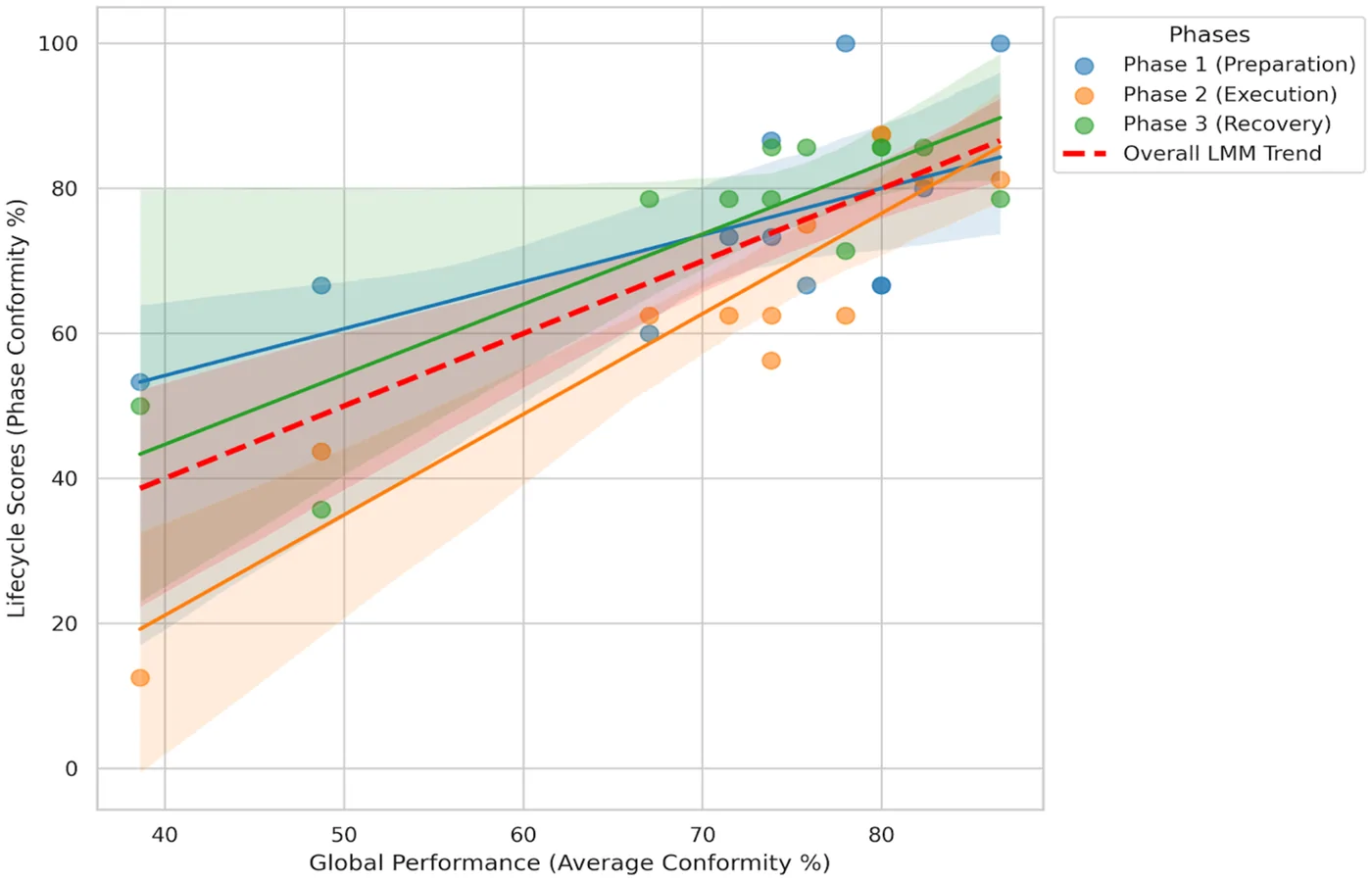
Scatter plot illustrating the relationship between global performance and technical scores at lifecycle scores across the Miluh-chagi movement phases. Correlation coefficient *r* = 0.90; *n* = 12 subjects (each performing 10 lifecycles).

Figure 1 shows the observation moment for contact of the foot with the boxing bag. The attacking leg and support leg are in obtuse flexion, the support leg foot is on tiptoe in an external rotation of less than 135°, and the site of contact is the sole.

**Figure 2:**
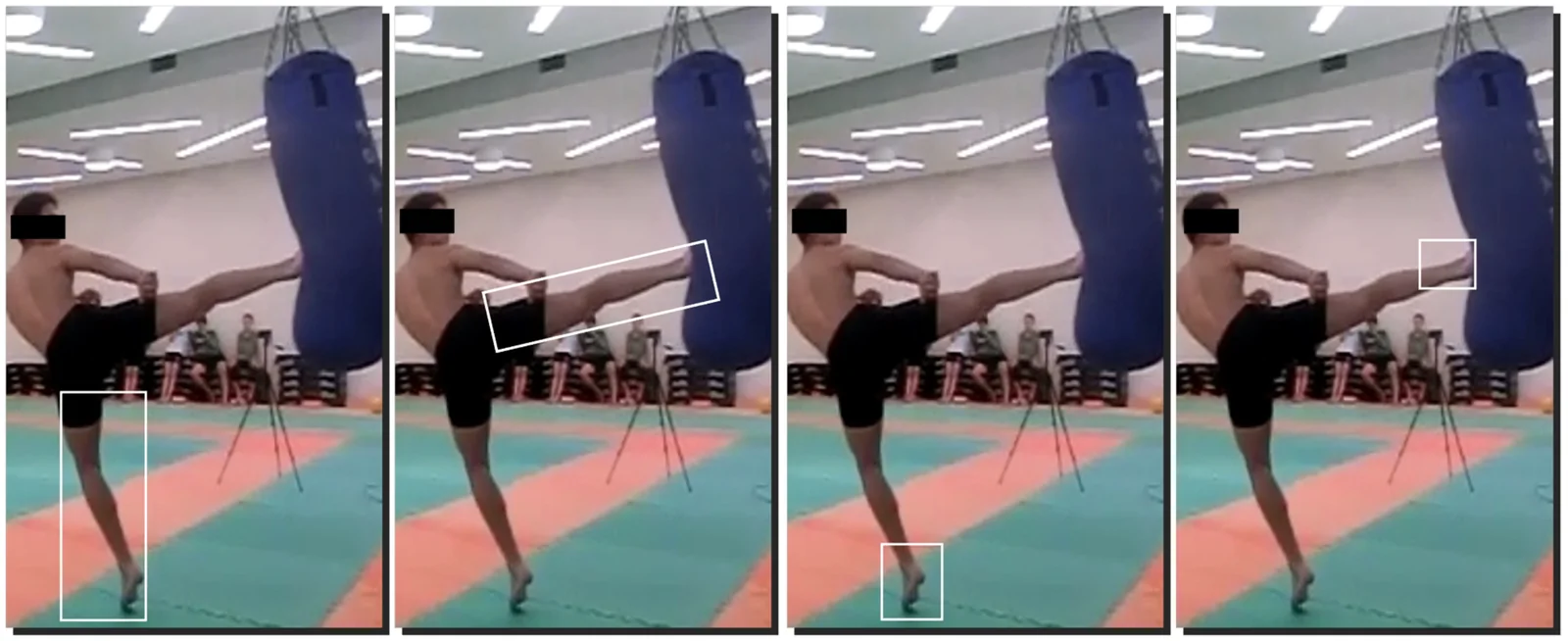
Event configuration framework for V4—contact moment.

The chart shows a strong linear trend (*p* < 0.001) where higher overall technical conformity corresponds to higher scores in each lifecycle phase of the Miluh-chagi movement. Based on the LMM, the data showed that Global Performance vs. Lifecycle Scores present a strong positive correlation across all three phases (Figure 2).

The correlation coefficient analysis results for the whole lifecycle of the kicks (Table 5) showed a perfect correlation between the “support leg's foot position and attacking leg's foot position” for the thigh extension moment (phase 3) (*r* = 1; *p* < 0.01).

**Table 5:** The whole lifecycle of ten kicks correlation coefficient analysis results.

Spearman's rho Phase 1 (position of):	Foot take-off (V1)								Knee up (V2)							
	AL1	AL2	SL1	SL2	H1	T1	La1	Ra1	AL3	AL4	SL3	SL4	H2	T2	La2	Ra2
Attacking leg	1.000								1.000							
Attacking leg foot	−0.135	1.000							0.090	1.000						
Support leg	**0**.364**	0.000	1.000						0.047	−0.114	1.000					
Support leg foot	0.068	0.092	0.021	1.000					**0**.241**	−0.048	−0.004	1.000				
Head	0.024	−0.142	−0.024	0.038	1.000				**0**.211*	−0.115	−0.032	0.020	1.000			
Trunk	0.035	−0.058	0.132	−0.096	0.173	1.000			−0.008	0.071	−0.156	0.093	0.043	1.000		
Left arm/forearm	0.055	0.078	0.160	0.028	**−0**.236**	−0.211	1.000		−0.091	−0.077	0.171	−0.040	0.025	−0.082	1.000	
Right arm/forearm	0.163	−0.234	0.039	−0.130	0.190	−0.197	−0.169	1.000	**0**.189*	−0.168	0.155	**0**.217*	**0**.216*	−0.038	**0**.184*	1.000
	Start leg extension (V3)								Contact moment (V4)							

Spearman's rho																
Phase 2 (position of):	AL5	AL6	SL5	SL6	H3	T3	La3	Ra3	AL7	AL8	SL7	SL8	H4	T4	La4	Ra4
Attacking leg	1.000								1.000							
Attacking leg foot	−0.162	1.000							−0.056	1.000						
Support leg	0.054	−0.175	1.000						−0.083	0.034	1.000					
Support leg foot	**−0**.276**	0.014	**0**.272**	1.000					**0**.190*	**−0**.324**	−0.046	1.000				
Head	**0**.337**	0.125	−0.128	**−0**.241**	1.000				**−0**.332**	**0**.181*	**−0**.208*	−0.108	1.000			
Trunk	0.147	**0**.186*	−0.007	−0.140	0.140	1.000			0.152	0.030	−0.023	**0**.238**	−0.158	1.000		
Left arm/forearm	0.146	0.093	**−0**.180*	−0.052	0.162	0.091	1.000		−0.171	−0.139	−0.150	0.136	0.088	0.103	1.000	
Right arm/forearm	−0.019	0.119	**−0**.286**	−0.063	**0**.330**	−0.017	**0**.298**	1.000	0.066	0.009	**−0**.358**	−0.006	0.002	0.030	0.123	1.000

Spearman's rho Phase 3 (position of):	Start leg flexion (V5)								Thigh extension (V6)							
	AL9	AL10	SL9	SL10	H5	T5	La5	Ra5	AL11	AL12	SL11	SL12	H6	T6	La6	Ra6
Attacking leg	1.000								1.000							
Attacking leg foot	0.129	1.000							**0**.336**	1.000						
Support leg	−0.101	0.175	1.000						0.156	**0**.329**	1.000					
Support leg foot	−0.024	**0**.324**	**0**.354**	1.000					**0**.336**	**1**.000**	**0**.329**	1.000				
Head	−0.145	0.108	**0**.396**	**0**.326**	1.000				**0**.273**	0.156	0.024	0.156	1.000			
Trunk	0.121	**−0**.214*	**−0228** *	**−0**.274**	**−0**.311**	1.000			0.036	**−0**.227*	−0.008	**−0**.227*	**−0**.257**	1.000		
Left arm/forearm	0.025	0.014	−0.114	−0.003	0.045	−0.129	1.000		**0**.204*	0.167	−0.014	0.167	−0.177	0.148	1.000	
Right arm/forearm	−0.117	0.049	**−0**.196*	0.048	0.103	−0.167	−0.149	1.000	0.126	0.204	0.171	**0**.204*	0.039	−0.108	0.115	1.000

Statistically significant correlations presented in bold.

* *p* < 0.05.

** *p* < 0.01.

## 4 Discussion

This study aimed to verify and analyze Taekwondo athletes' technical performance indicators during the lifecycles of ten kicks, namely *Miluh-chagi*. All the participants were black belt Taekwondo practitioners. The (OSTIP-C) (2) and a panel of Taekwondo experts were used as evaluators. The results of our study suggest that the OSTIP-C has good reliability and precision in analyzing motor competence and produced results that are in line with those of previous papers (2, 9, 15, 37). The adolescent athlete sample comprised five female (41.7%) and seven male (58.3%) Taekwondo practitioners, each of whom performed 10 pushing kick techniques. The athletes were not statistically significantly different in terms of age and number of years practicing Taekwondo (*p* > 0.05). The males revealed a statistically significant height difference [*t*(10) = 3.958; *p* < 0.05], with a moderate ES, and weight [*t*(10) = 2.950; *p* < 0.05], with a small ES. Overall, these findings from several studies support the usefulness of the OSTIP-C as a reliable tool for the systematic analysis of technical performance indicators in taekwondo athletes.

The coefficient of variation was calculated to verify the reliability among the evaluators and showed very good reliability. The results shown in Table 1 confirm the reliability of the evaluators (25, 26, 28, 38). The intra-rater reliability was 0.958 (*p* < 0.01), which is lower than that of Sousa et al. (2) and Barrientos et al. (39), demonstrating excellent to perfect reliability. Nonetheless, the result obtained in this study is still considered excellent compared to those of other studies (19–21). This study showed an inter-rater reliability of 0.946 (*p* < 0.01), as in previous research (2, 9). According to past studies (20), values above 0.800 are considered good in terms of evaluator reliability. All the results in this study, which were obtained using the OSTPI-C tool, showed excellent to perfect or nearly perfect reliability.

In phase 1, the foot take-off, attacking leg, contact foot, and trunk positions presented the highest mean values throughout the entire kick lifecycle. These values align with those of previous studies (15, 39). Only the position of the attacking leg foot showed a statistically significant difference (*p* < 0.01); this unexpected finding can be explained by the athletes' morpho-functional muscle structure, speed, impact accuracy, or skill level. The athletes showed statistically significant differences in the position of the attacking leg foot [*t*(118) = 4.811; *p* < 0.01], with a large ES (29, 38). The positions of the head and attacking leg foot showed a large ES, but the difference was not statistically significant (*p* > 0.05) (29, 38). These results are consistent with those reported by Sousa et al. (2). During the knee-up moment, the support leg was in an obtuse flexed position, the thigh of the attacking leg was in acute flexion, and the leg position was highest. These results align with those of published studies (9, 37, 40). Consequently, these results reinforce the relevance of proper body alignment and limb positioning during the initial phase of the kicking cycle to optimize technical performance in taekwondo.

In contrast, the left arm and forearm were in an obtuse flexion position at a higher level, which was not observed in previous studies (9). These results can be explained by the Taekwondo black belt level (level of expertise of the athletes in this study). Only the position of the attacking leg's foot showed a statistically significant difference, in contrast to previous papers that did not find a significant difference (2, 9). Differences in the Taekwondo weight categories or the black belt variable could explain this. The results showed that the athletes had statistically significant differences in the position of the attacking leg foot [*t*(118) = 4.264; *p* < 0.01], with a large ES. The head position and right arm and forearm position showed a very large ES, but the difference was not statistically significant (*p* > 0.05), which is in line with the findings of several published studies (29–31, 38). The foot positioning at take-off was found to be crucial for optimizing force transfer and impact accuracy, which aligns with previous findings on Taekwondo biomechanics (9). At the start of the leg extension moment (phase 2), the support leg is in extension, the foot is facing outward in external rotation less than 180°, and the right arm is in extension, while the forearm flexion position is higher throughout the technique's lifecycle. These results were expected based on previously published studies (2, 9, 37). Therefore, these findings highlight the importance of appropriate foot positioning and limb coordination during the extension phase, which may contribute to optimizing force transmission and technical effectiveness in taekwondo kicking performance.

This study found a trivial to moderate ES for the attacking leg position, with a large ES of 0.845 (|*d*| ≥ 0.80) (29–31, 38). During the contact moment, the highest values were observed for the support leg conduct criteria; this leg is in an obtuse flexion position, and in the attacking leg, the tip of the foot is in flexion of the toes. Meanwhile, the contact area between the foot and the target showed a higher reliability index than expected, based on previous studies that reported a perfect reliability index (2, 9, 37). The result also showed that the support leg, left arm and forearm positions present a large ES of 0.846 and 0.958 (|*d*| ≥ 0.80), respectively. These results could be explained by the fact that, in this study, a different kick technique was analysed, performed differently and with different execution trajectories. In this phase, none of the conduct or aggregate criteria showed statistically significant differences. These results were expected based on published studies (9, 32, 37). Also, these findings emphasize the relevance of lower-limb positioning and foot–target contact mechanics during the impact phase, which appear to contribute to the effectiveness and control of the kicking technique in taekwondo.

The support leg, foot, arm extension, and forearm flexion positions were found to be critical for enhancing balance and correct foot contact, aligning with previous findings from Taekwondo analyses. At the start of the leg flexion moment (phase 3), there were statistically significant differences in the positions of the support leg and attacking leg foot [*t*(118) = 3.272; *p* < 0.01]), with a score of 1.889, which corresponds to a large ES (|*d*| ≥ 0.80); this indicates that the position of the support leg foot is facing out after an external rotation of up to 90°. The trunk and right arm and forearm positions showed a large ES (1.050 and 1.213, respectively; |*d*| ≥ 0.80), which is in line with the findings of previous papers (9, 29, 30, 38). These thigh extension results are in line with those of previous studies that also showed large ESs (9, 32). There were no statistically significant differences in any conduct or aggregate criteria. The position of the trunk, the left and right arms, and the forearm showed moderate ES of 0.564, 0.597 and 0.502 (|*d*| = 0.50–0.79), respectively. These results aligned with those of previous studies and supported the assumption of the methodology (9, 29, 30, 38). The non-significant differences in the right arm positioning may be due to individual variability in upper body stabilization strategies among the athletes.

The correlation coefficient values for all variables were less than 0.85, which indicates that there were no multicollinearity problems (41). A correlation coefficient analysis was carried out using Spearman's rho to assess the strength of the correlations (25, 26, 32, 37, 41–43). In our study, there was a moderate negative correlation between the positions of the support leg's foot and the attacking leg's foot at the moment of contact. In addition, there were correlations between the head and attacking leg positions and the support leg position and between the trunk and head positions at the start of the leg flexion moment. A moderate correlation was found 1) between the positions of the attacking leg's foot and attacking leg during foot take-off; 2) between the positions of the head and attacking leg, right arm and forearm and head, and left arm and forearm at the start of the leg extension moment; 3) between the positions of the support leg's foot and attacking leg's foot and support leg, and between the positions of the head and support leg and support leg's foot at the start of the leg flexion moment; 4) between the positions of the attacking leg's foot and attacking leg, and between the positions of the support leg and attacking leg's foot during thigh extension; and 5) between the positions of the support leg's foot and attacking leg, and support leg. Our study showed a perfect correlation (*r* = 1; *p* < 0.01) between the positions of the support leg's foot and the attacking leg during the thigh extension moment.

LMM and the data showed that Global Performance vs. Lifecycle Scores present a strong positive correlation across all three phases. As the Global Performance (average conformity) increases, the scores for individual phases also rise significantly. Phases 1 (preparation) and 3 (recovery) show the most consistent alignment with the overall trend. Phase 2 (execution) shows slightly higher variance at lower performance levels but tightens as global conformity reaches the 89%–100% range. Conduct and aggregate criteria show a small to moderate negative correlation reported, with *r* = 0.181; *p* < 0.05 and *r* = 0.396; *p* < 0.01, respectively. Overall, the fixed effect of the model shows a steady linear progression across the technical lifecycle. These results were not expected based on previous and published studies (9, 37), which reported perfect reliability for the contact area of the foot with the target (boxing bag). Our result could be explained by the fact that we analyzed a different technique with different characteristics and trajectory lines compared with previous studies (2, 9, 37). This study may have shortcomings because it did not measure the lower limbs, which some authors claim is an important variable that must be measured (5, 6, 8, 10, 11). Nonetheless, our findings provide valuable insights that can enhance the observational skills of coaches, trainers, and athletes when analyzing Taekwondo kicking techniques, specifically Miluh-chagi.

### 4.1 Strengths

This study has at least two strengths. First, the panel of observers was composed of five evaluators, including one world-level Taekwondo expert (7th DAN black belt) with more than 35 years of experience, one European expert (6th DAN black belt) with over 30 years of experience, two Portuguese higher national-level experts (5th DAN black belt) with more than 20 years of experience, and one Portuguese middle national-level expert (4th DAN black belt) with 15 years of experience. Second, we used an observational tool (the OSTIP-C) as an alternative to the traditional way (eyeballing) of observing the athlete's motor performance. All the training stakeholders can easily use this instrument.

### 4.2 Limitations and future research directions

The limitations of the study were: (1) the sample size; (2) the lack of measurements for the lower limbs (which could be important for combat Taekwondo strategy); (3) the data on the lifecycle of the ten kicks were not considered in terms of different weight categories or black belt ranks (level of athlete's expertise); and (4) a single-session design that may affect ecological validity and cause fatigue (e.g., kyorugi differs from static bag work by requiring recognition reaction time and constant biomechanical adjustments. This dynamic environment shifts performance from simple execution to complex perception-action coupling).

Future studies could (1) measure the leg length, which could be used to analyze the reliability of the motor behavior of the athlete's Taekwondo kicking technique; (2) integrate motion capture analysis for validation; and (3) perform kinematic analysis or sensor-based tracking to obtain deeper insights. Although the expert evaluators were trained, potential biases in subjective scoring cannot be ruled out.

### 4.3 Practical applications

The OSTIP-C is a specialized instrument developed for the systematic assessment of technical performance across eight primary Taekwondo kick. Furthermore, it functions as a supplement to traditional video analysis, enabling the precise identification to discern both performance critical errors and the success factors.

The results of this study highlight the early phases of the pushing kick—specifically foot take-off, knee up, and start leg flexion—as the most technically variable and therefore the most relevant for targeted training. Coaches can use this information to design focused drills that isolate these phases, emphasizing hip flexion speed, segmental coordination, and postural control. By concentrating training efforts on these initial moments, practitioners can reduce execution variability and improve overall kicking efficiency in adolescent athletes. As this is a critical moment characterized by considerable variability, plyometric training and dynamic exercises are recommended.

The high reliability of the OSTIP-C observational system demonstrates its value as a practical tool for daily training environments. Coaches and evaluators can incorporate this structured methodology to monitor technical progression, identify recurring execution errors, and provide objective, consistent feedback. Additionally, the strong relationship observed between support-leg alignment and attacking-leg mechanics reinforces the importance of balance, proprioception, and support-leg strength training, which can be integrated into warm-ups or complementary conditioning sessions to enhance kicking stability and performance.

## 5 Conclusions

This study demonstrated that the observational methodology supported by the OSTIP-C system is a reliable and precise tool for analyzing the technical performance indicators of the pushing kick in adolescent Taekwondo athletes. The high intra- and inter-rater reliability values confirm that trained evaluators can consistently identify and score the biomechanical phases of the kick, reinforcing the validity of structured observational systems in combat sports.

The significant differences observed in the foot take-off, knee up, and start leg flexion phases highlight these early moments of the movement as the most variable and technically demanding. These phases appear to be critical for the coordination and efficiency of the kicking action, suggesting that they should be prioritized in technical training and performance monitoring. In contrast, the absence of significant differences in the later phases—start leg extension, contact moment, and thigh extension—indicates greater execution stability once the movement reaches its peak velocity and alignment.

The identified correlation patterns, particularly the perfect association between support leg and attacking leg foot positions during thigh extension, emphasize the interdependence of lower-limb segments in achieving optimal kicking mechanics. These findings contribute to a deeper understanding of the motor structure of the pushing kick and provide evidence-based guidance for coaches seeking to refine athletes' technical execution.

Overall, this study supports integrating validated observational tools into Taekwondo training environments and highlights the importance of focusing on the initial phases of the kick to enhance motor competence and performance outcomes in adolescent athletes.

## Data Availability

The raw data supporting the conclusions of this article will be made available by the authors, without undue reservation.
